# Neighborhood Deprivation and Risks of Autoimmune Disorders: A National Cohort Study in Sweden

**DOI:** 10.3390/ijerph16203798

**Published:** 2019-10-09

**Authors:** Xinjun Li, Jan Sundquist, Tsuyoshi Hamano, Kristina Sundquist

**Affiliations:** 1 Center for Primary Health Care Research, Lund University, 205 02 Malmö, Sweden; jan.sundquist@med.lu.se (J.S.); kristina.sundquist@med.lu.se (K.S.); 2 Department of Family Medicine and Community Health, Department of Population Health Science and Policy, Icahn School of Medicine at Mount Sinai, New York, NY 10001, USA; 3 Center for Community-based Healthcare Research and Education (CoHRE), Department of Functional Pathology, School of Medicine, Shimane University, Izumo 690-2406, Japan; 4 Department of Sports Sociology and Health Sciences, Kyoto Sangyo University, Kyoto 520-0461, Japan; thamano@cc.kyoto-su.ac.jp; 5 Department of Functional Pathology, Shimane University School of Medicine, Izumo 690-2406, Japan

**Keywords:** autoimmune disorders, neighborhood deprivation, risk factors, Sweden

## Abstract

*Background:* No study to date, as far as we know, has analyzed the potential effect of neighborhood-level deprivation on autoimmune disorders (ADs), when adjusted for individual-level characteristics. *Methods:* A total of 5.4 million individuals from 8363 neighborhoods, comprising the whole Swedish population (ages 25–74 years), were followed for the period 1 January 2000, until admission due to diagnosis of ADs during the period of the study, or the conclusion of the study (31 December 2010). We used a neighborhood deprivation index, constructed from variables such as low education, low income, unemployment, and social welfare assistance, to assess the level of neighborhood deprivation. Multilevel logistic regression was used in the analysis with individual level characteristics at the first level and level of neighborhood deprivation at the second level. *Results:* A significant association between level of neighborhood deprivation and ADs was found. The crude odds were 1.32 (95% confidence interval 1.27–1.36) for those residing in the high-deprived neighborhoods compared to those living in low-deprivation neighborhoods. In the full model, where individual level characteristics were taken into account, the odds of ADs were 1.18 (1.14–1.22) in the most deprived neighborhoods. Certain Ads—angiitis hypersensitive (5.14), ankylosing spondylitis (1.66), celiac disease (1.65), Crohn’s disease (1.21), diabetes mellitus type 1 (1.45), Graves’s disease (1.13), Hashimoto thyroiditis (1.51), psoriasis (1.15), rheumatoid arthritis (1.15), sarcoidosis (1.20), and systemic sclerosis (1.27)—remained significantly associated with high level of neighborhood deprivation after adjustment for the individual-level variables. *Conclusion:* This study is the largest to date analyzing the potential influence of neighborhood deprivation on ADs. Our results indicate that neighborhood deprivation may affect risk of ADs, independent of individual level sociodemographic characteristics. For health care policies, both individual and neighborhood level approaches seem to be of importance.

## 1. Introduction

Autoimmune disorders (ADs) are a heterogeneous group of between 70 to 80 specific disorders that affect approximately 3% to 8% of the populations in the United States [1] and the Nordic Countries [2]. The number of individuals affected by ADs is increasing; between 14.7 million to 23.5 million people have Ads, according to previous studies from the U.S. [1,3]. ADs consist of a broad range of disorders, including both autoimmune and inflammatory disorders. Some ADs, such as type 1 diabetes mellitus, may be present as early as in the first decade of life. However, symptoms of most ADs typically become apparent in the third to sixth decade of life [3]. The nature of the etiologies of ADs is largely unknown. A number of risk factors for ADs have been previously mentioned. These include female sex, family history of ADs [4], and socio–economic factors [5,6,7,8] including education [9].

Recently, interest has greatly increased concerning the contextual effects of neighborhood deprivation on different health outcomes [10], and the use of multilevel modeling has helped to disentangle the effects of neighborhood characteristics from individual-level risk factors [11,12]. To cite an example, if people living in the same neighborhood share the same socioeconomic environment, access to healthcare, norms settings, and lifestyles, they may characterize a common level of health beyond individual characteristics. Thus, together with individual-level sociodemographic factors, neighborhood-level factors may also increase the risk of many diseases. For example, neighborhood deprivation is associated with several cardiovascular risk factors; it has been shown to be an important independent risk factor for coronary heart disease and other cardiovascular diseases [13,14,15,16,17], psychiatric disorders [18], and certain specific types of ADs [19,20,21]. There is also a link between cardiovascular diseases and ADs [22]. No study to date, as far as we know, has analyzed the potential effect of neighborhood-level deprivation on ADs, when adjusted for individual-level characteristics.

Our first aim with this study was to investigate whether there is an association between neighborhood deprivation and incidence of ADs. The neighborhoods were derived from small geographic units covering all Sweden. Socio–economic status or level of deprivation of these units was classified as high, middle or low, based on a previously used neighborhood deprivation index (based on low educational status, low income, unemployment, and social welfare assistance). Our second aim was to investigate whether this possible difference stays the same after accounting for individual-level sociodemographic characteristics, and hospitalization for chronic obstructive pulmonary disease and/or alcohol-related diseases.

## 2. Materials and Methods

The data used in this study were retrieved from registers located at the Center for Primary Health Care Research at Lund University. The dataset used for this study incorporated information on the entire national population that comprised a period of 40 years. It included hospital inpatient and outpatient data for the whole population during the study period. In addition, the dataset incorporated population-wide documentation concerning concomitant factors such as geographical region and socio–economic status. We used the primary diagnoses for ADs in the Swedish In-Patient Register (data are available between 1964–2010) and Out-Patient Register (2001–2010). Additional linkages were carried out on national census data (to gather individual socio–economic status, geographical region of residence, and the other socio–demographic data), The Registry of Cause of Death (to identify date and cause of death; data are available between 1961–2010), and the Immigration Registry (to identify date of emigration). All linkages were performed via use of a personal national ID number that is assigned to each person in Sweden, either at birth or at immigration to the country, and is used throughout their lifetime. To ensure anonymity, a serial number replaced this number for each individual.

The follow-up period commenced on 1 January 2000 and continued until first registration for ADs, death, emigration, or the conclusion of the study period (31 December 2010). Prior to being enrolled into the study, individuals who had previously (i.e., between 1997–1999) been diagnosed with any autoimmune disorders (n = 33,528) were removed from the study in order to exclude pre-existing cases. The study population comprised a nationwide sample of 2,698,818 men and 2,679,542 women aged 25–74 years.

### 2.1. Outcome Variable

Diagnoses were reported according to the tenth version of the International Classification of Diseases (ICD–10), classified in 43 autoimmune conditions of diseases.

### 2.2. Individual Variables

Individual variables were defined at the start of the follow-up and included sex, age at the start of the study, marital status, family income, educational attainment, immigration status, geographical region, and mobility of the subjects. Hospitalization for chronic lower respiratory diseases, alcoholism, and related liver disease were defined during the study period.

### 2.3. Sex. Male and Female

Age. Age was divided into 10-year categories. Age was used as a continuous variable in the adjustment.

Marital status. Individuals were classified as married/cohabitating or single (never married, widowed or divorced).

Family income by quartile. Statistics Sweden provided the information on family income (2000) that was obtained from the Total Population Register. We used the distribution to calculate empirical quartiles.

Educational attainment. Educational attainment was classified as level of education ≤ 9 years, 10–12 years, or > 12 years.

### 2.4. Immigration Status: (1) Born in Sweden and (2) Born outside Sweden

Region of residence: Large cities (Stockholm, Gothenburg, Malmö), southern Sweden, and northern Sweden.

Move: Length of years spent living in a neighborhood, categorized as moved (lived in neighborhood < 5 years) or not moved (lived in neighborhood > 5 years). The rationale for adjusting for mobility is because mobility may have an influence on the exposure, i.e., neighborhood deprivation.

Hospitalization for chronic obstructive pulmonary disease (COPD) was identified in the Hospital Registry (ICD-10 = J40–J49). Patients’ COPD status in the Hospital Registry was individually linked to their ADs status via usage of a serial number (see above). Hospitalization for alcoholism and related liver disease was also identified in the Hospital Registry according to the International Classification of Diseases (ICD) codes (ICD–10 = F10 and K70).

Neighborhood Deprivation Index: We used a summary measure to characterize neighborhood-level deprivation. Deprivation indicators used by past studies were identified to characterize neighborhood environments; we then used a principal components analysis to select deprivation indicators in the Swedish national database [14]. The following four variables were selected for those aged 25–74: low educational status (< 10 years of formal education); low income (income from all sources, including that from interest and dividends, defined as less than 50% of individual median income); unemployment (not employed, excluding full-time students, those completing compulsory military service, and early retirees); and social welfare assistance. Each of the four variables loaded on the first principal component with similar loadings (+ 0.47 to + 0.53) and explained 52% of the variation between these variables. Neighborhood deprivation was assessed in 2000.

As supplied to us by Statistics Sweden, a z score was calculated for each neighborhood, defined based on the geographic units SAMS (Small Areas Market Statistics). The z scores were weighted by the coefficients for the eigenvectors, and then summed to create an index [23]. The index was categorized into three groups: low (index below one standard deviation (SD) from the mean), moderate (index within one SD of the mean), and high (index above one SD from the mean). Higher scores reflected more deprived neighborhoods. A total of 8363 SAMS units were included in our study (Supplementary Table S1).

### 2.5. Statistical Analysis

Age-standardized cumulative incidence was calculated by direct age standardization using 10-year age groups specific to women or men, with the entire Swedish population of women or men in the year 2000 as the standard population. For the outcome variables, we used multilevel (hierarchical) logistic regression models with incidence proportions (the proportion of adults who became cases among those entered into the study time interval). Analyses were performed using MLwiN, version 2.27. First, a null model was calculated to determine the variance among neighborhoods. A neighborhood model was subsequently then calculated that only included neighborhood-level deprivation to determine the crude odds of ADs incidence by level of neighborhood deprivation (model 1). We next calculated a model that included neighborhood-level deprivation and sex, age (model 2), and eight individual-level sociodemographic variables, added simultaneously to the model (model 3) Finally, a full model was calculated that included neighborhood-level deprivation, all the individual-level sociodemographic variables, and hospitalization for COPD and/or hospitalization for alcoholism and related liver disease, which were added simultaneously to the model (model 4). These full models tested whether neighborhood-level deprivation was significantly associated with ADs when adjusted for the individual characteristics, and whether there were differential effects of neighborhood-level deprivation on ADs across individual characteristics [24], i.e., cross-level interactions.

Random effects: The between-neighborhood variance was estimated both with and without a random intercept. If it was larger than 1.96 times the standard error then it was regarded as significant; this concurs with the precedent established in prior studies [18,25].

There were no meaningful cross-level interactions or effect modifications shown in the test for cross-level interactions between the individual-level variables and neighborhood-level deprivation on odds of ADs. The analyses were therefore performed with both sexes combined.

### 2.6. Ethical Considerations

The Ethics Committee of Lund University, Sweden approved this study (2012/795).

## 3. Results

Table 1 shows population sizes and neighborhood characteristics for the year 2000 by neighborhood-level deprivation. In total the number of neighborhoods was 8363 (Supplementary Table S1). Of the total population, 25% (low), 59% (moderate), and 16% (high) lived in the three types of neighborhood. In the follow-up period, there were 82,587 (1.5%) of individuals who were diagnosed with ADs. There were more women than men with ADs in the patient population. Age-adjusted ADs incidence rates were 13.0 per 1000 in neighborhoods with low deprivation, in neighborhoods with moderate deprivation the figure was 15.9 per 1000, and in neighborhoods with high deprivation, it was 17.0 per 1000. A similar pattern of higher ADs with each increasing level of neighborhood-level deprivation was shown across all ten individual-level variable categories, hospitalization for COPD, and hospitalization for alcoholism and related liver disease. Significance tests to make comparisons across neighborhoods are shown in Table 2 and Supplementary Table S2. All categories indicated a gradient effect across level of neighborhood deprivation (Table 2 and Supplementary Table S2).

Table 3 show the ICD codes and the total number of patients with autoimmune diseases. The largest diagnostic groups were diabetes mellitus type 1 (14.6%), and rheumatoid arthritis (9.4%). Age-adjusted incidence rates of diabetes mellitus type 1 were 24.6 per 10,000 in neighborhoods with low deprivation, with moderate deprivation, the figure was 37.9 per 10,000, and in neighborhoods with high deprivation, it was 47.7 per 10,000. For rheumatoid arthritis, the rates were 19.2, 24.4, and 23.2 per 10,000 individuals, respectively.

For individuals living in a high versus low deprivation neighborhood, the odds ratio (OR) of ADs was 1.32 (1.27–1.36) in the crude model (Table 4). High neighborhood-level deprivation remained significantly associated with ADs when adjusted for the ten individual-level variables, hospitalization for COPD, and hospitalization for alcoholism and related liver disease (OR = 1.18; 95% CI, 1.14–1.22). The highest odds of ADs were found in individuals who were male, never married, widowed, or divorced, had the lowest educational attainment, had moved, were hospitalized for COPD, and/or were hospitalized for alcoholism and related liver disease. Individuals who lived in Southern Sweden or immigrants had lower odds of ADs. Interestingly, the highest odds ratios were found among individuals with middle level family income.

Table 5 shows the results for specific ADs. For certain Ads—angiitis hypersensitive, ankylosing spondylitis, celiac disease, Crohn’s disease, diabetes mellitus type 1, Graves’ disease, Hashimoto thyroiditis, psoriasis, rheumatoid arthritis, sarcoidosis, and systemic sclerosis—the high level of neighborhood deprivation remained significantly associated with those ADs after adjustment for the individual-level variables. Supplementary Figure S1 shows a forest plot of key findings for specific ADs in high level of neighborhood deprivation compared with low level of neighborhood deprivation.

We performed an additional analysis using logistic regression models and Cox regression models, and the results were almost identical (Supplementary Tables S3 and S4).

The association between each of the four deprivation indicators in the neighborhood deprivation index and ADs are shown in Supplementary Table S5.

The between-neighborhood variance (i.e., the random intercept) was over 1.96 times the standard error in all models; this indicates that there were major differences in ADs between neighborhoods after taking into account the neighborhood-level variable as well as the individual-level variables. The neighborhood-level variable accounted for 22% of the between-neighborhood variance in the null model (see Table 4). The explained variance was 51% after we included the individual level variables.

## 4. Discussion

The main findings of this study are that the odds of ADs are higher among individuals living in deprived neighborhoods than individuals living in affluent neighborhoods. This difference remained significant, even after we adjusted for the ten individual-level sociodemographic variables, hospitalization for COPD, and hospitalization for alcoholism and related liver disease. The present study represents a novel contribution as no previous neighborhood study has focused on several specific types of ADs [19,20,21]. For specific Ads—angiitis hypersensitive, ankylosing spondylitis, celiac disease, Crohn’s disease, diabetes mellitus type 1, Graves’ disease, Hashimoto thyroiditis, psoriasis, rheumatoid arthritis, sarcoidosis, and systemic sclerosis—the high level of neighborhood deprivation remained significantly associated with those ADs after adjustment for the individual-level variables.

Individual-level sociodemographic factors, which indicate socioeconomic inequality [5,6,7,8,9], have been reported to be associated with risk of ADs. However, the causal pathways between neighborhood socioeconomic deprivation and poor health outcomes are not fully understood; several possible mechanisms could lie behind our findings. One possible mediator could be psychological stress [26,27,28,29,30], due to littered and unsafe environments, vandalism, isolation/alienation, and violent crime in deprived neighborhoods [18]. It is possible that the lack of safe environments reduces the possibility to exercise; this may aggravate a healthy lifestyle. In addition, socio–cultural norms, regarding smoking and physical activity, could vary between neighborhoods and impact the health of the residents and the risk for ADs. A Swedish study showed that physical inactivity, obesity, and smoking were more common among individuals that lived in deprived neighborhoods than among individuals that lived in affluent neighborhoods [13]. A study from Denmark found that neighborhood deprivation is associated with participation in a population-based health check, in which increasing neighborhood deprivation was associated with decreasing participation. The authors suggested that there is a need to develop preventative health checks tailored to deprived neighborhoods [31].

Studies have shown that risk factors, such as neighborhood socio–economic status [21], neighborhood income [20], and geographical variation [32], are associated with ADs. Although several studies of ADs have used neighborhood level socioeconomic status as a proxy for individual socioeconomic status, another study that represents one of the few studies on this topic found that, after using hierarchical multilevel modelling and controlling for individual socioeconomic status and other covariates, only the association between neighborhood socioeconomic status and ADs remained significant. A Canadian study revealed a significant neighborhood influence on the incidence of Crohn’s disease and ulcerative colitis [33]. In the present study, the ORs decreased after adding hospitalization for COPD, alcoholism, and related liver disease to the models, which indicates that the neighborhood influences on ADs could be partly mediated by risk factors, such as smoking and alcohol.

Living in neighborhoods with low social deprivation can be a basis for isolation from health-promoting environments (e.g., safe places to conduct physical exercise and proper housing) and social services. Associations between neighborhood characteristics and different health outcomes were found to be inconsistent in a 2001 Lancet study where wealthy nations were compared [34]. This finding suggests that neighborhood determinants of health are multifaceted. These determinants can include the likes of access to health care, education, and social services. In the U.S., access to these types of services is uneven, as the impact of income inequalities on health are more distinct [35]. A study from the U.S. revealed demographic and environmental factors that affect the incidence and cause of SLE by studying geographical variation and clusters in mortality from the disease [32]. According to the census data, clusters with elevated mortality had higher poverty rates and/or greater concentrations of ethnic Hispanics than those with lower mortality [36]. The spatial variation in poverty, Hispanic ethnicity, and solar radiation partly explained the strong pattern of geographical clustering of mortality from SLE in the U.S. [36].

There are some limitations to our study. For example, in studies concerning neighborhood effects on health, selective residential mobility can cause compositional neighborhood differences [37]. Selective residential mobility is the propensity for people to move to neighborhoods that have characteristics that match their individual characteristics (for example, the propensity for people with low socioeconomic status to move to low socio–economic status neighborhoods). However, we made an adjustment for family income; this enhanced our chances to establish the differences between compositional and contextual effects on ADs. It cannot be ruled out, however, that residual confounding exists. For example, socio–economic status cannot be measured precisely and completely and it is possible that there were confounders that we were not able to control for entirely, including smoking and food habit. Our study also has many strengths. The large cohort included the vast majority of patients with ADs in Sweden during the period of the study; this increased the generalizability of our results. Another strength is the use of personal identification numbers, which enabled us to follow individuals in different registers, for example, the Immigration Register, which permitted calculation of exact risk time in the additional analyses. Furthermore, the Swedish Total Population Register is highly complete, with very few missing data. Finally, our use of multilevel modeling enabled us to separate neighborhood-level and individual-level effects and permitted us to consider both fixed and random effects in the analyses.

## 5. Conclusions

Neighborhood deprivation seems to have an independent contribution to ADs, although causality cannot be proven. The neighborhood-level and individual-level variables may cumulatively load against individuals so that the most at-risk individuals would be those who have both individual- and neighborhood-level risk factors. These findings raise important clinical and public health concerns, and indicate that both individual- and neighborhood-level approaches are important in health care policies. In addition, possible pathways from neighborhood deprivation to AD need to be studied in detail in order to address the etiology behind neighborhood differences in AD risk.

## Figures and Tables

**Table 1:** Distribution of population, number of autoimmune disorder events, and age-standardized incidence (per 1000) by neighborhood-level deprivation.

Individual Variables	Autoimmune Disorder Events						
	Population		N. of Events		Incidence Rates by Neighborhood Deprivation		
	No.	(%)	No.	(%)	Low	Moderate	High
Total population (%)	5,378,360				1,316,124 (25%)	3,180,078 (59%)	882,158 (16%)
Total events			82,587		13.0	15.9	17.0
Sex							
Male	2,698,818	50.2	36,968	44.8	11.7	14.3	15.2
Female	2,679,542	49.8	45,619	55.2	14.3	17.4	18.7
Age (years)							
25–34	1,210,432	22.5	11,301	13.7	8.4	9.5	9.9
35–44	1,205,477	22.4	12,694	15.4	8.7	10.9	11.9
45–54	1,213,003	22.6	17,370	21.0	12.1	14.8	16.3
55–64	1,020,846	19.0	20,283	24.6	16.7	20.5	22.7
65–74	728,602	13.5	20,939	25.4	26.2	28.8	31.4
Family income							
Low income	1,345,419	25.0	20,263	24.5	13.8	16.9	16.9
Middle-low income	1,345,830	25.0	23,800	28.8	14.7	17.9	19.1
Middle-high income	1,344,824	25.0	21,286	25.8	13.6	15.9	16.7
High income	1,342,287	25.0	17,238	20.9	11.6	13.2	13.7
Marital status							
Married/cohabiting	2,773,647	51.6	42,637	51.6	12.3	14.9	15.4
Never married, Widowed, or divorced	2,604,713	48.4	39,950	48.4	14.6	17.2	18.5
Immigrant status							
Sweden	4,624,597	86.0	72,280	87.5	13.2	16.1	17.7
Other countries	753,763	14.0	10,307	12.5	11.5	14.4	15.3
Educational attainment							
≤ 9 years	849,993	15.8	20,589	24.9	14.9	16.9	17.6
10–12 years	552,451	10.3	8823	10.7	14.9	17.6	18.5
> 12 years	3,975,916	73.9	53,175	64.4	12.5	15.1	16.1
Region of residence							
Large cities	2,720,123	50.6	40,711	49.3	12.9	16.2	17.0
Southern Sweden	1,789,149	33.3	27,601	33.4	13.4	15.4	16.6
Norther Sweden	869,088	16.2	14,275	17.3	13.0	16.2	17.9
Move							
Not moved	3,778,649	70.3	62,069	75.2	12.9	15.8	16.9
Moved	1,599,711	29.7	20,518	24.8	13.2	16.4	17.3
Hospitalization of chronic lower respiratory disease							
No	5,259,280	97.8	77,417	93.7	12.6	15.4	16.3
Yes	119,080	2.2	5170	6.3	36.8	39.3	38.7
Hospitalization of alcoholism and related liver disease							
No	5,261,431	97.8	79,175	95.9	12.8	15.6	16.6
Yes	116,929	2.2	3412	4.1	27.6	28.9	27.4

**Table 2:** Baseline characteristics of events of autoimmune disorders in neighborhoods.

Individual variables	Neighborhood Deprivation						*p*-Value
	Low (*n* = 17,111)		Moderate (*n* = 50,496)		High (*n* = 14,980)		
	No.	%	No.	%	No.	%	
Gender							0.263
Male	7565	44.2	22,669	44.9	6734	45.0	
Female	9546	55.8	27,827	55.1	8246	55.0	
Age (years)							< 0.001
25–34	2386	13.9	6727	13.3	2188	14.6	
35–44	2743	16.0	7564	15.0	2387	15.9	
45–54	3757	22.0	10,610	21.0	3003	20.0	
55–64	4304	25.2	12,526	24.8	3453	23.1	
65–74	3921	22.9	13,069	25.9	3949	26.4	
Family income							< 0.001
Low income	2505	14.6	12,512	24.8	5246	35.0	
Middle-low income	3899	22.8	15,084	29.9	4817	32.2	
Middle-high income	4759	27.8	13,268	26.3	3259	21.8	
High income	5948	34.8	9632	19.1	1658	11.1	
Marital status							< 0.001
Married/cohabiting	10,133	59.2	26,128	51.7	6376	42.6	
Never married, Widowed, or divorced	6978	40.8	24,368	48.3	8604	57.4	
Immigrant status							< 0.001
Sweden	15,542	90.8	45,579	90.3	11,159	74.5	
Other countries	1569	9.2	4917	9.7	3821	25.5	
Educational attainment							< 0.001
≤ 9 years	2492	14.6	13,203	26.1	4894	32.7	
10–12	1435	8.4	5459	10.8	1929	12.9	
>12 years	13,184	77.0	31,834	63.0	8157	54.5	
Region of residence							< 0.001
Large cities	10,855	63.4	22,211	44.0	7645	51.0	
Southern Sweden	4312	25.2	18,664	37.0	4625	30.9	
Northern Sweden	1944	11.4	9621	19.1	2710	18.1	
Move							< 0.001
Not moved	12,951	75.7	38,544	76.3	10,574	70.6	
Moved	4160	24.3	11,952	23.7	4406	29.4	
Hospitalization of chronic lower respiratory disease							< 0.001
No	16,224	94.8	47,355	93.8	13,838	92.4	
Yes	887	5.2	3141	6.2	1142	7.6	
Hospitalization of alcoholism and related liver disease							< 0.001
No	16,558	96.8	48,506	96.1	14,111	94.2	
Yes	553	3.2	1990	3.9	869	5.8	

**Table 3:** Number of autoimmune disorder events, and age-standardized incidence (per 10,000 individuals) by neighborhood-level deprivation.

Autoimmune Disorder	ICD–10 Code	N. of Events		Incidence Rates by Neighborhood Deprivation		
		No.	%	Low	Moderate	High
Addison disease	E27.1, E27.2	593	0.41	1.22	1.05	1.10
Amyotrophic lateral sclerosis	G12.2	2047	1.46	3.90	3.80	3.68
Angiitis hypersensitive	M31.0	29	0.02	0.02	0.06	0.09
Ankylosing spondylitis	M45, M08.1	1021	0.76	1.44	1.97	2.34
Autoimmune hemolytic anemia	D59.0,D59.1	33	0.02	0.05	0.06	0.09
Behcet disease	M35.2	98	0.05	0.14	0.14	0.39
Celiac disease	K90.0	326	0.25	0.43	0.65	0.71
Chorea minor	I02	3	0.00	0.00	0.01	0.01
Chronic rheumatic heart disease	I05-I09	1176	0.75	2.09	1.96	3.16
Crohn disease	K50	6663	4.92	10.96	12.78	13.10
Dermatitis Herpetiformis	L13.0	22	0.01	0.05	0.03	0.05
Diabetes mellitus type I	E10	19,516	14.61	24.63	37.94	47.74
Discoid lupus erythematosus	L93.0	70	0.06	0.08	0.16	0.10
Giant-cell arteritis	M31.5,M31.6	2860	2.05	5.36	5.33	5.21
Glomerluar nephritis chronic	N00,N01	1570	1.13	2.67	2.94	3.22
Glomerular nephritis acute	N03	399	0.29	0.75	0.75	0.69
Grave disease	E05.0,E05.5	3139	2.21	5.56	5.75	6.57
Guillain–Barre Syndrome	G61.0	1065	0.73	2.24	1.89	1.93
Hashimoto thyroiditis	E03.5,E03.8,E03.9,E06.3	2045	1.59	2.58	4.12	4.48
Immune thrombocytopenic purpura	D69.3	985	0.70	1.80	1.81	1.95
Localized scleroderma	L94.0	69	0.05	0.13	0.13	0.14
Lupoid hepatitis	K75.4	362	0.27	0.65	0.69	0.65
Multiple sclerosis	G35	4363	3.21	7.90	8.33	7.64
Myasthenia gravis	G70.0	813	0.59	1.47	1.53	1.51
Pemphigoid	L12 (not L12.2)	351	0.25	0.59	0.64	0.80
Pemphigus	L10 (not L10.3 and L10.5)	119	0.06	0.28	0.16	0.36
Pernicious anemia	D51.0	192	0.13	0.28	0.34	0.52
Polyarteritis nodosa	M30.0	124	0.08	0.24	0.22	0.26
Polymyalgia rheumatic	M35.3	3561	2.71	5.47	7.03	6.87
Polymyositis/dermatomyositis	M33	523	0.38	0.88	0.99	1.05
Primary biliary cirrhosis	K74.3	554	0.42	0.89	1.08	1.04
Psoriasis	L40	2382	1.80	3.62	4.66	4.80
Reiter disease	M02.3	55	0.04	0.09	0.11	0.09
Rheumatic fever	I00,I01	56	0.05	0.09	0.12	0.06
Rheumatoid arthritis	M05, M06, M08.0, M08.2	12,343	9.40	19.25	24.42	23.17
Sarcoidosis	D86	1953	1.51	2.98	3.92	3.57
Sjögren syndrome	M35.0	549	0.41	0.89	1.05	1.10
Systemic lupus erythematosus	M32	1306	0.98	2.12	2.53	2.51
Systemic sclerosis	M34	774	0.58	1.22	1.51	1.51
Takayasus disease	M31.4	81	0.05	0.13	0.14	0.24
Thrombotic thrombocytop	M31.1	100	0.08	0.15	0.21	0.14
Ulcerative colitis	K51	7387	5.41	13.20	14.04	13.43
Wegener granulomatosis	M31.3	910	0.68	1.50	1.75	1.75
All		82,587	61.14	130.01	158.79	169.81

**Table 4:** Odds ratios (OR) and 95% confidence intervals (CI) for autoimmune disorders; Results of multi-level logistic regression models.

Variables	Model 1			Model 2			Model 3			Model 4			
	OR	95% CI		OR	95% CI		OR	95% CI		OR	95% CI		*p*-Value
Neighborhood-level variable (ref. Low)													
Moderate	1.23	1.19	1.26	1.20	1.17	1.23	1.13	1.10	1.16	1.13	1.10	1.16	<0.001
High	1.32	1.27	1.36	1.31	1.26	1.35	1.20	1.16	1.25	1.18	1.14	1.22	<0.001
Age				1.03	1.03	1.03	1.03	1.03	1.03	1.03	1.03	1.03	<0.001
Sex to male (ref. female)				1.23	1.20	1.25	1.21	1.18	1.24	1.23	1.20	1.25	<0.001
Family income (ref. High income)													
Low income							1.23	1.19	1.27	1.20	1.16	1.24	<0.001
Middle-low income							1.30	1.26	1.34	1.26	1.22	1.30	<0.001
Middle-high income							1.19	1.15	1.22	1.17	1.14	1.20	<0.001
Marital status (ref. Married/co-habiting)													
Never married, widowed, or divorced							1.16	1.14	1.19	1.13	1.11	1.15	<0.001
Immigrant status (ref. Born in Sweden)							0.85	0.83	0.88	0.85	0.83	0.88	<0.001
Education attainment (ref. > 12 years)													
≤ 9 years							1.13	1.10	1.16	1.12	1.09	1.15	<0.001
10–12 years							1.15	1.11	1.19	1.13	1.09	1.17	<0.001
Region of residence (ref. Large cities)													
Southern Sweden							0.95	0.92	0.97	0.96	0.93	0.98	<0.001
Northern Sweden							0.98	0.95	1.01	0.99	0.96	1.02	0.549
Move (ref. Not moved)							1.07	1.05	1.10	1.06	1.03	1.08	<0.001
Hospitalization of chronic lower respiratory disease (ref. No)										2.08	2.00	2.17	<0.001
Hospitalization of alcoholism and related liver disease (ref. No)										1.74	1.65	1.83	<0.001
Variance (S.E.)	0.029 (0.003)			0.021 (0.003)			0.018 (0.003)			0.018 (0.003)			
Explained variance (%)	22			43			51			51			

Note: Model 1: crude model; Model 2: adjusted for age and sex; Model 3: adjusted for age, sex, family income, marital status, country of birth, education, region of residence, and move. Model 4: adjusted for age, sex, family income, marital status, country of birth, education, region of residence, move, and hospitalization of chronic low respiratory disease and alcoholism and related liver disease.

**Table 5:** Odds ratios (OR) and 95% confidence intervals (CI) for autoimmune disorders *.

Autoimmune Disorders	Neighborhood Deprivation							*p*-Value
	Low, Reference	Moderate			High			
	OR	OR	95% CI		OR	95% CI		
Addison disease	1.00	0.86	0.70	1.04	0.94	0.72	1.22	0.627
Amyotrophic lateral sclerosis	1.00	0.95	0.85	1.06	1.01	0.88	1.17	0.852
Angiitis hypersensitive	1.00	3.28	0.75	14.34	5.14	1.05	25.19	0.044
Ankylosing spondylitis	1.00	1.35	1.14	1.60	1.66	1.35	2.04	< 0.001
Autoimmune hemolytic anemia	1.00	0.78	0.32	1.91	1.17	0.40	3.40	0.771
Behçet disease	1.00	1.01	0.59	1.76	1.69	0.92	3.13	0.092
Celiac disease	1.00	1.45	1.07	1.97	1.65	1.13	2.40	0.010
Chorea minor	1.00	-	-	-				
Chronic rheumatic heart disease	1.00	0.88	0.76	1.02	1.12	0.94	1.34	0.223
Crohn disease	1.00	1.14	1.07	1.21	1.21	1.11	1.31	< 0.001
Dermatitis Herpetiformis	1.00	0.54	0.20	1.46	0.75	0.20	2.73	0.657
Diabetes mellitus type I	1.00	1.25	1.21	1.31	1.45	1.38	1.52	< 0.001
Discoid lupus erythematosus	1.00	1.79	0.92	3.49	1.13	0.45	2.83	0.800
Giant-cell arteritis	1.00	0.93	0.85	1.02	0.94	0.83	1.06	0.308
Glomerluar nephritis chronic	1.00	1.01	0.89	1.15	1.07	0.91	1.26	0.425
Glomerular nephritis acute	1.00	0.93	0.73	1.18	0.86	0.61	1.20	0.361
Graves’ disease	1.00	1.04	0.95	1.13	1.13	1.01	1.27	0.032
Guillain–Barré Syndrome	1.00	0.87	0.75	1.01	0.97	0.80	1.19	0.789
Hashimoto thyroiditis	1.00	1.53	1.35	1.72	1.51	1.30	1.76	< 0.001
Immune thrombocytopenic purpura	1.00	0.91	0.78	1.06	0.95	0.77	1.17	0.642
Localized scleroderma	1.00	0.84	0.47	1.51	0.90	0.41	1.96	0.786
Lupoid hepatitis	1.00	1.04	0.80	1.34	0.97	0.68	1.38	0.857
Multiple sclerosis	1.00	1.06	0.99	1.14	1.05	0.95	1.16	0.354
Myasthenia gravis	1.00	0.97	0.81	1.15	0.99	0.79	1.25	0.939
Pemphigoid	1.00	0.87	0.66	1.15	0.98	0.70	1.38	0.916
Pemphigus	1.00	0.50	0.32	0.78	0.83	0.49	1.39	0.476
Pernicious anemia	1.00	0.92	0.62	1.35	1.24	0.79	1.95	0.358
Polyarteritis nodosa	1.00	0.86	0.56	1.33	1.08	0.61	1.91	0.786
Polymyalgia rheumatic	1.00	1.10	1.00	1.20	1.10	0.98	1.23	0.097
Polymyositis/dermatomyositis	1.00	1.16	0.93	1.44	1.28	0.96	1.70	0.095
Primary biliary cirrhosis	1.00	1.08	0.87	1.34	1.00	0.75	1.34	0.989
Psoriasis	1.00	1.17	1.06	1.31	1.15	1.00	1.32	0.047
Reiter disease	1.00	1.16	0.59	2.29	1.00	0.39	2.55	0.996
Rheumatic fever	1.00	1.45	0.75	2.84	0.72	0.24	2.11	0.543
Rheumatoid arthritis	1.00	1.18	1.13	1.24	1.15	1.08	1.22	< 0.001
Sarcoidosis	1.00	1.25	1.11	1.40	1.20	1.02	1.40	0.024
Sjögren syndrome	1.00	1.23	0.99	1.53	1.25	0.94	1.66	0.120
Systemic lupus erythematosus	1.00	1.16	1.01	1.33	1.12	0.93	1.35	0.224
Systemic sclerosis	1.00	1.26	1.05	1.52	1.27	1.00	1.62	0.051
Takayasus disease	1.00	1.09	0.61	1.94	1.81	0.92	3.59	0.088
Thrombotic thrombocytop	1.00	1.22	0.73	2.04	0.83	0.40	1.75	0.626
Ulcerative colitis	1.00	1.03	0.97	1.09	1.02	0.94	1.10	0.700
Wegener granulomatosis	1.00	1.12	0.94	1.32	1.24	0.99	1.55	0.057

Note: * Full adjusted model.

## Supplementary Materials

The following are available online at https://www.mdpi.com/1660-4601/16/20/3798/s1, Table S1: Population sizes and neighborhood characteristics by neighborhood-level deprivation; Table S2: Baseline characteristics of study population in neighborhoods; Table S3: Odds ratio (OR) and 95% confidence intervals (CI) for autoimmune disorders; Results of logistics regression models; Table S4: Hazard ratios (HR) and 95% confidence intervals (CI) for autoimmune disorders; Results of Cox regression models; Table S5: Odds ratio (OR) and 95% confidence intervals (CI) for autoimmune disorders for each of the four deprivation indicators in the neighborhood deprivation index *; Figure S1: Adjusted Odds ratios (OR) for autoimmune disorders for individuals living a high level of neighborhood deprivation compared with low level of neighborhood deprivation. Whiskers are 95% confidence intervals.
