# Supplementary material for: Neighborhood Deprivation and Risks of Autoimmune Disorders: A National Cohort Study in Sweden
Source: Int J Environ Res Public Health. 2019 Oct 9;16(20):3798. doi: 10.3390/ijerph16203798 (PMC6843782; doi:10.3390/ijerph16203798)
Supplement: Supplementary file 1 [file ijerph-16-03798-s001.pdf]

Supplementary

Table S1. Population sizes and neighborhood characteristics by neighborhood-level deprivation.

Individual Variables	Neighborhood Deprivation		
	Low	Moderate	High
Number of neighborhoods	1898	5069	1396
Number of people			
Men	648737	1598838	451243
Women	667387	1581240	430915
Percent of people			
Men	24.0	59.2	16.7
Women	24.9	59.0	16.1
Neighborhood deprivation index			
Range	−3.0 to −1	−1 to 1	> 1 to 10.7
Mean	−1.47 ± 0.32	−0.12 ± 0.54	2.47 ± 1.65
Median	−1.42	−0.17	1.84

Table S2. Baseline characteristics of study population in neighborhoods.

Individual Variables	Neighborhood deprivation						*p*-value
	Low (n=1316124)		Moderate (n=3180078)		High (n=882158)		
	No.	%	No.	%	No.	%	
Gender							< 0.001
Male	648,737	49.3	1,598,838	50.3	451,243	51.2	
Female	667,387	50.7	1,581,240	49.7	430,915	48.8	
Age (years)							< 0.001
25-34	283,917	21.6	706,280	22.2	220,235	25.0	
35-44	313,738	23.8	691,575	21.7	200,164	22.7	
45-54	311,337	23.7	717,733	22.6	183,933	20.9	
55-64	257,486	19.6	611,297	19.2	152,063	17.2	
65-74	149,646	11.4	453,193	14.3	125,763	14.3	
Family income							< 0.001
Low income	201,198	15.3	805,251	25.3	338,970	38.4	
Middle–low income	276,554	21.0	834,747	26.2	234,529	26.6	
Middle–high income	342,589	26.0	815,914	25.7	186,321	21.1	
High income	495,783	37.7	724,166	22.8	122,338	13.9	
Marital status							< 0.001
Married/cohabiting	773,899	58.8	1,613,298	50.7	386,450	43.8	
Never married, Widowed, or divorced	542,225	41.2	1,566,780	49.3	495,708	56.2	
Immigrant status							< 0.001
Sweden	1,183,153	89.9	2,833,395	89.1	608,049	68.9	
Other countries	132,971	10.1	346,683	10.9	274,109	31.1	
Educational attainment							< 0.001
≤ 9 years	109,937	8.4	537,798	16.9	202,258	22.9	
10–12	96,587	7.3	337,664	10.6	118,200	13.4	
> 12 years	1,109,600	84.3	2,304,616	72.5	561,700	63.7	
Region of residence							< 0.001
Large cities	852,911	64.8	1,398,287	44.0	468,925	53.2	
Southern Sweden	315,772	24.0	1,201,989	37.8	271,388	30.8	
Nothern Sweden	147,441	11.2	579,802	18.2	141,845	16.1	
Move							< 0.001
Not moved	922,918	70.1	2,,282,408	71.8	573,323	65.0	
Moved	393,206	29.9	897,670	28.2	308,835	35.0	
Hospitalization of chronic lower respiratory disease							< 0.001
No	1,294,352	98.3	3,108,674	97.8	856,254	97.1	

Yes	21,772	1.7	71,404	2.2	25,904	2.9	
Hospitalization of alcoholism and related liver disease							< 0.001
No	1,297,071	98.6	3,112,765	97.9	851,595	96.5	
Yes	19,053	1.4	67,313	2.1	30,563	3.5	

Table S3. Odds ratio (OR) and 95% confidence intervals (CI) for autoimmune disorders; Results of logistics regression models.

Variables	Model 1			Model 2			Model 3			Model 4			
	OR	95% CI		OR	95% CI		OR	95% CI		OR	95% CI		*p*-value
**Neighborhood-level variable (ref. Low)**													
Moderate	1.23	1.20	1.25	1.20	1.17	1.22	1.13	1.11	1.15	1.12	1.10	1.14	< 0.001
High	1.31	1.28	1.34	1.31	1.28	1.34	1.20	1.18	1.23	1.18	1.15	1.21	< 0.001
Age				1.03	1.03	1.03	1.03	1.03	1.03	1.03	1.03	1.03	< 0.001
Gender to male (ref. female)				1.22	1.21	1.24	1.21	1.19	1.22	1.22	1.21	1.24	< 0.001
Family income (ref. High income)													
Low income							1.24	1.21	1.26	1.21	1.18	1.23	< 0.001
Middle-low income							1.31	1.28	1.34	1.27	1.25	1.30	< 0.001
Middle-high income							1.20	1.18	1.23	1.18	1.16	1.21	< 0.001
Marital status (ref. Married/co-habiting)													
Never married, widowed, or divorced							1.18	1.17	1.20	1.15	1.13	1.16	< 0.001
Immigrant status (ref. Born in Sweden)							0.85	0.83	0.87	0.85	0.83	0.87	< 0.001
Education attainment (ref. > 12 years)													
≤ 9 years							1.13	1.11	1.15	1.12	1.10	1.14	< 0.001
10–12 years							1.15	1.12	1.17	1.13	1.10	1.15	< 0.001
Region of residence (ref. Large cities)													
Southern Sweden							0.95	0.94	0.97	0.96	0.95	0.98	< 0.001
Northern Sweden							0.99	0.97	1.01	1.00	0.98	1.02	0.786
Move (ref. Not moved)							1.06	1.04	1.08	1.04	1.02	1.06	< 0.001
Hospitalization of chronic lower respiratory disease (ref. No)										2.04	1.98	2.10	< 0.001
Hospitalization of alcoholism and related liver disease (ref. No)										1.72	1.66	1.78	< 0.001

Model 1: crude model; Model 2: adjusted for age and gender; Model 3: adjusted for age, gender, family income, marital status, country of birth, education, region of residence, and move; Model 4: adjusted for age, gender, family income, marital status, country of birth, education, region of residence, move, and hospitalization of chronic low respiratory disease and alcoholism and related liver disease

Table S4. Hazard ratios (HR) and 95% confidence intervals (CI) for autoimmune disorders; Results of Cox regression models.

Variables	Model 1			Model 2			Model 3			Model 4			
	HR	95% CI		HR	95% CI		HR	95% CI		HR	95% CI		*p*-value
Neighborhood-level variable (ref. Low)													
Moderate	1.23	1.21	1.25	1.20	1.18	1.22	1.12	1.10	1.14	1.12	1.10	1.14	< 0.0001
High	1.33	1.30	1.36	1.34	1.31	1.37	1.21	1.18	1.24	1.18	1.16	1.21	< 0.0001
Age				1.03	1.03	1.03	1.03	1.03	1.03	1.03	1.03	1.03	< 0.0001
Gender to male (ref. female)				1.20	1.18	1.21	1.18	1.16	1.20	1.20	1.18	1.21	< 0.0001
Family income (ref. High income)													
Low income							1.27	1.24	1.30	1.24	1.21	1.27	< 0.0001
Middle-low income							1.33	1.31	1.36	1.29	1.27	1.32	< 0.0001
Middle-high income							1.21	1.18	1.23	1.19	1.16	1.21	< 0.0001
Marital status (ref. Married/cohabiting)													
Never married, widowed, or divorced							1.20	1.19	1.22	0.88	0.86	0.89	< 0.0001
Immigrant status (ref. Born in Sweden)							0.87	0.86	0.89	0.88	0.86	0.89	< 0.0001
Education attainment (ref. > 12 years)													
≤ 9 years							1.15	1.13	1.17	1.14	1.12	1.16	< 0.0001
10–12 years							1.14	1.12	1.17	1.12	1.10	1.15	< 0.0001
Region of residence (ref. Large cities)													
Southern Sweden							0.95	0.93	0.96	0.96	0.94	0.97	< 0.0001
Northern Sweden							0.98	0.96	1.00	0.99	0.97	1.01	0.451
Move (ref. Not moved)							1.07	1.06	1.09	1.06	1.04	1.07	< 0.0001
Hospitalization of chronic lower respiratory disease (ref. No)										2.13	2.07	2.19	< 0.0001
Hospitalization of alcoholism and related liver disease (ref. No)										1.82	1.75	1.88	< 0.0001

Model 1: crude model; Model 2: adjusted for age and gender; Model 3: adjusted for age, gender, family income, marital status, country of birth, education, region of residence, and move; Model 4: adjusted for age, gender, family income, marital status, country of birth, education, region of residence, move, and hospitalization of chronic low respiratory disease and alcoholism and related liver disease

Table S5. Odds ratio (OR) and 95% confidence intervals (CI) for autoimmune disorders for each of the four deprivation indicators in the neighborhood deprivation index *.

Neighborhood-level Variables	High	Moderate			Low		
	OR	OR	95% CI		OR	95% CI	
Neighbhorhood income	1.00	1.02	1.00	1.04	1.05	1.03	1.08
Neighbhorhood education	1.00	1.11	1.09	1.13	1.18	1.16	1.21
Neighbhorhood social welfare	1.00	1.07	1.05	1.09	1.15	1.12	1.17
Neighborhood employment	1.00	1.10	1.08	1.12	1.17	1.14	1.19

*Adjusted for full model.

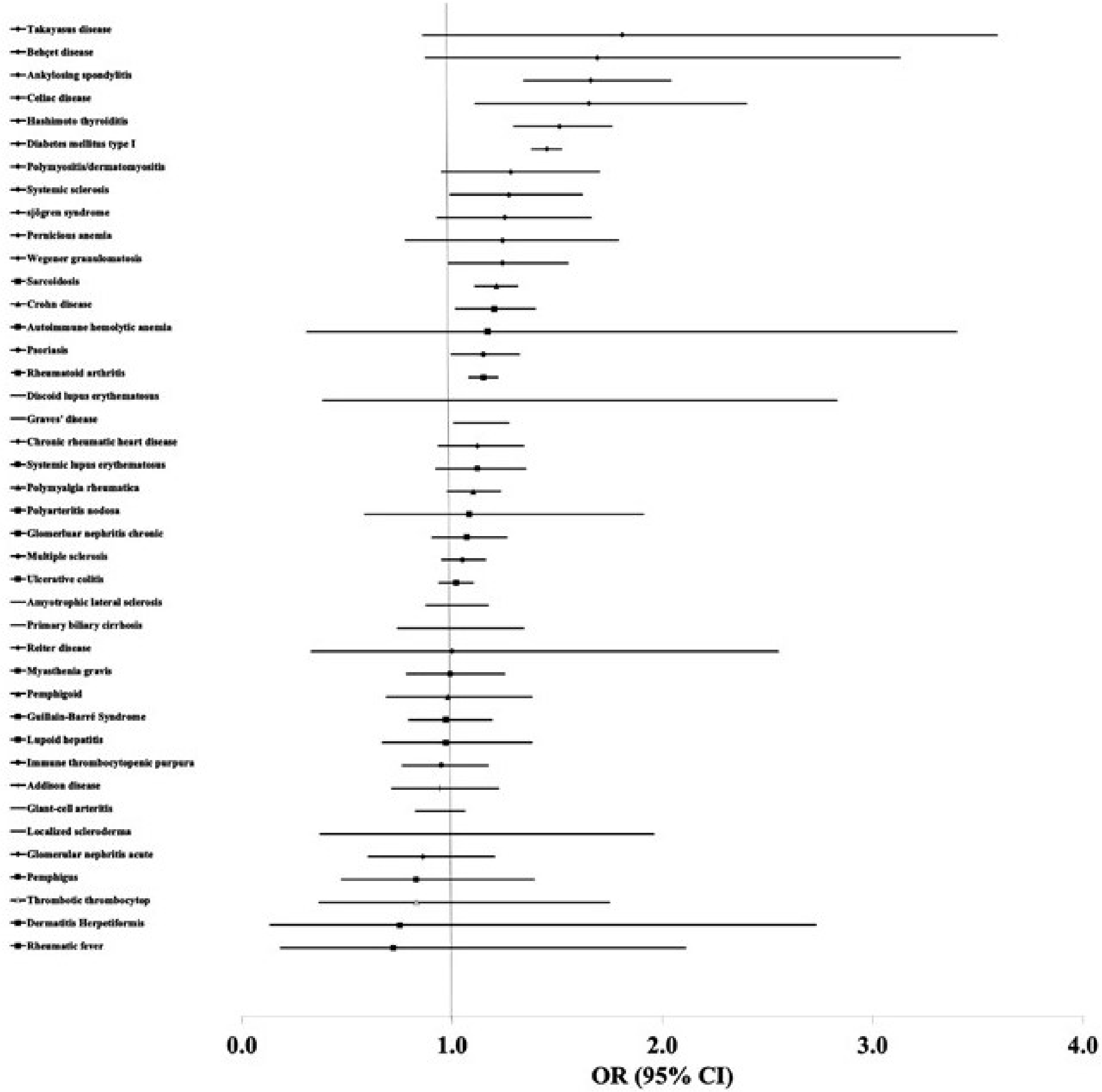

Figure S1. Adjusted Odds ratios (OR) for autoimmune disorders for individuals living a high level of neighborhood deprivation compared with low level of neighborhood deprivation. Whiskers are 95% confidence intervals. *Data was based on the Table 5, OR of angiitis hypersensitive (OR=5.14, 95% 1.05-25.19) was not shown in the figure.
